# Supplementary figures and images for: Critical multi-stranded approach for determining the ecological values of diatoms in unique aquatic ecosystems of anthropogenic origin
Source: PeerJ. 2019 Dec 5;7:e8117. doi: 10.7717/peerj.8117 (PMC6899344; doi:10.7717/peerj.8117)

## PE

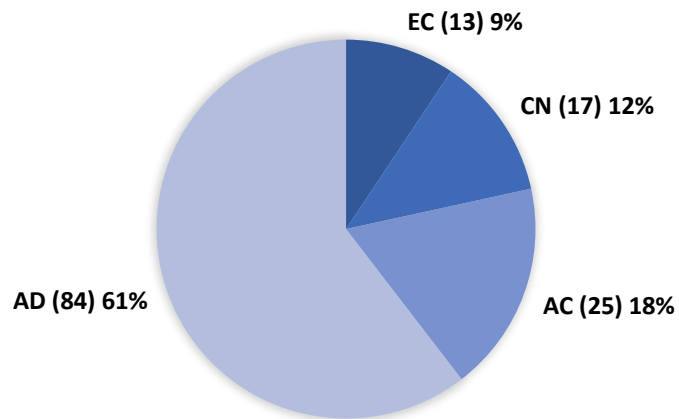

## LE

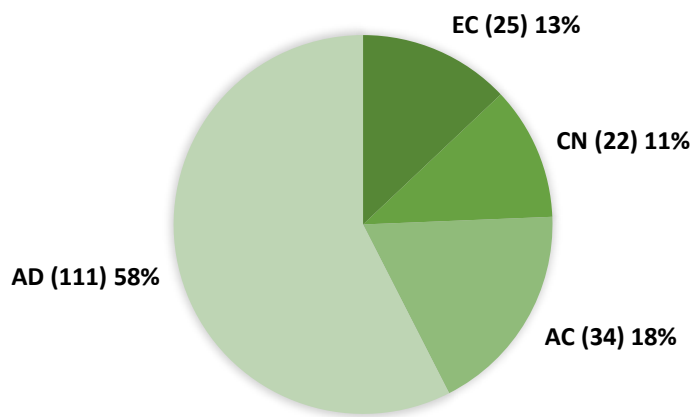

## BO

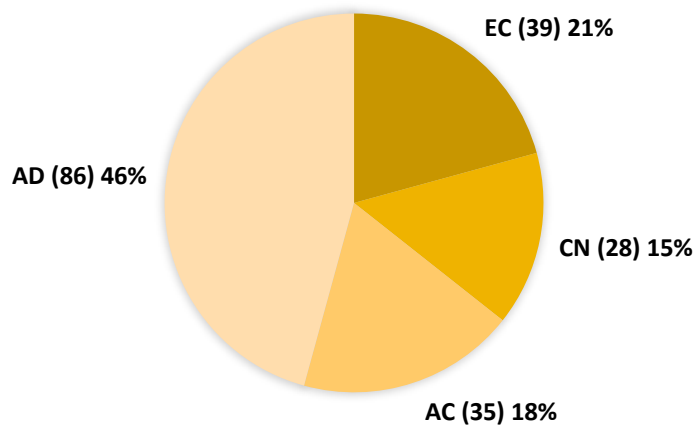

Supplement: Supplemental Information 1 — PE, Pełczyska complex; LE, Łęczyca reservoir; BO, Bogdałów reservoir; EC, euconstant taxa, CN, constant taxa; AC, accessory taxa; AD, accidental taxa. The number of species is shown in brackets. [file peerj-07-8117-s001.pdf]
