# Supplementary material for: Critical multi-stranded approach for determining the ecological values of diatoms in unique aquatic ecosystems of anthropogenic origin
Source: PeerJ. 2019 Dec 5;7:e8117. doi: 10.7717/peerj.8117 (PMC6899344; doi:10.7717/peerj.8117)
Supplement: Supplemental Information 2 — A. pH requirements. B. Salinity. C. Nitrogen uptake. D. Oxygen requirements. E. Saprobity. F. Trophic state. G. Moisture aerophily [file peerj-07-8117-s002.pdf]

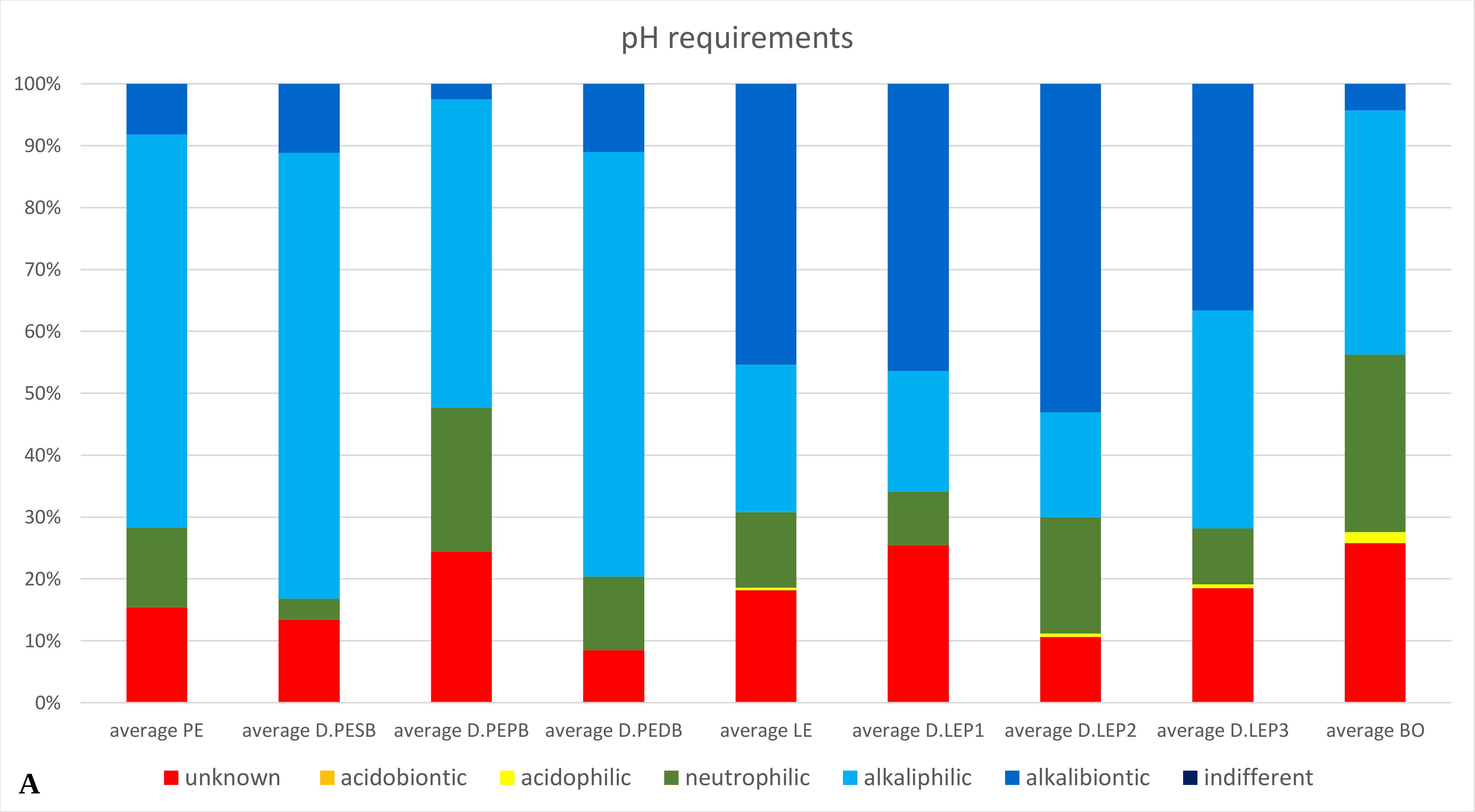
pH requirements
100%
90%
80%
70%
60%
50%
40%
30%
20%
10%
0%
average PE
average D.PESB
average D.PEPB
average D.PEDB
average LE
average D.LEP1
average D.LEP2
average D.LEP3
average BO
unknown
acidobiontic
acidophilic
neutrophilic
alkaliphilic
alkalibiontic
indifferent
A

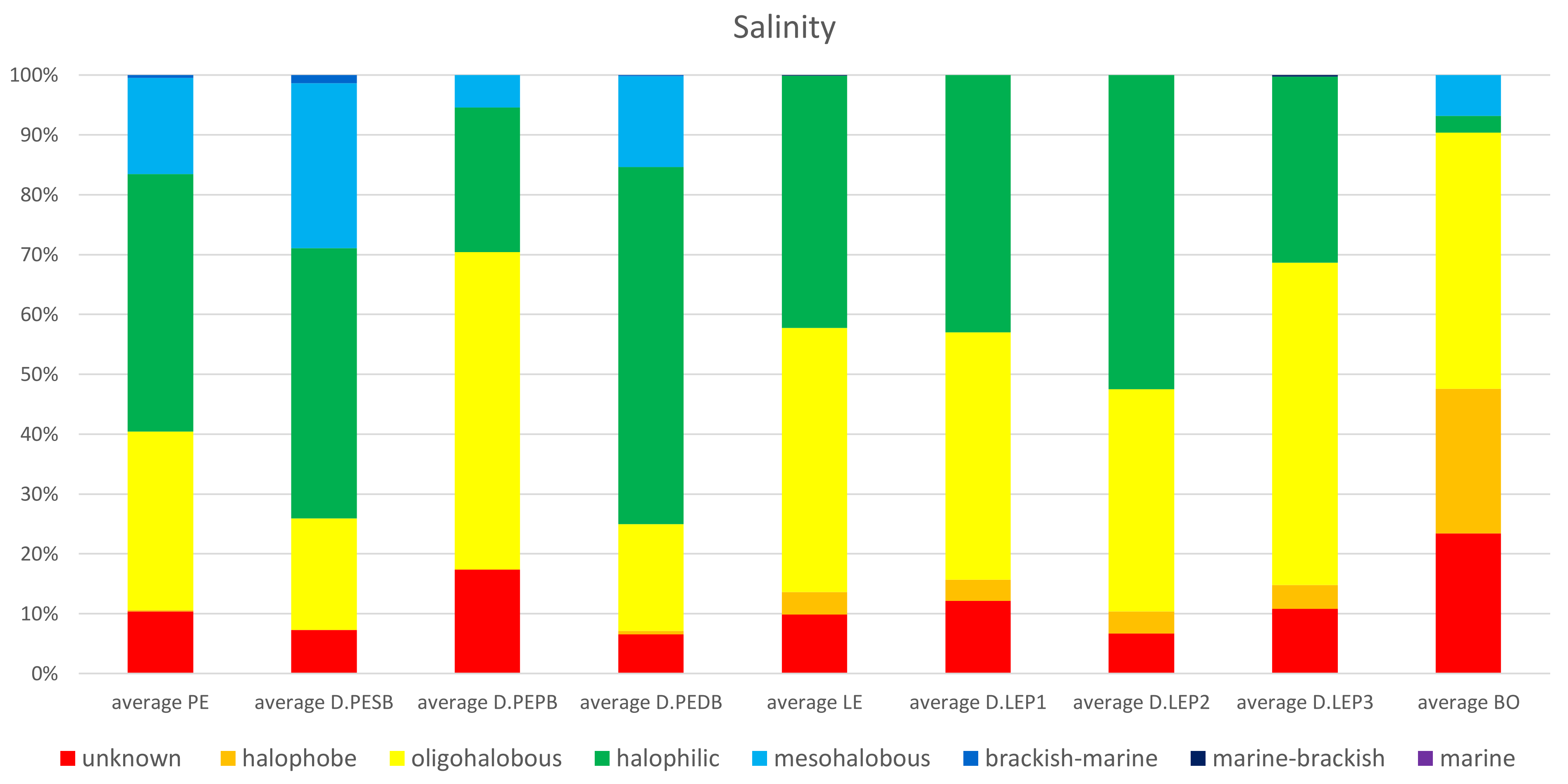
Salinity
100%
90%
80%
70%
60%
50%
40%
30%
20%
10%
0%
average PE
average D.PESB
average D.PEPB
average D.PEDB
average LE
average D.LEP1
average D.LEP2
average D.LEP3
average BO
unknown
halophobe
oligohalobous
halophilic
mesohalobous
brackish-marine
marine-brackish
marine

B

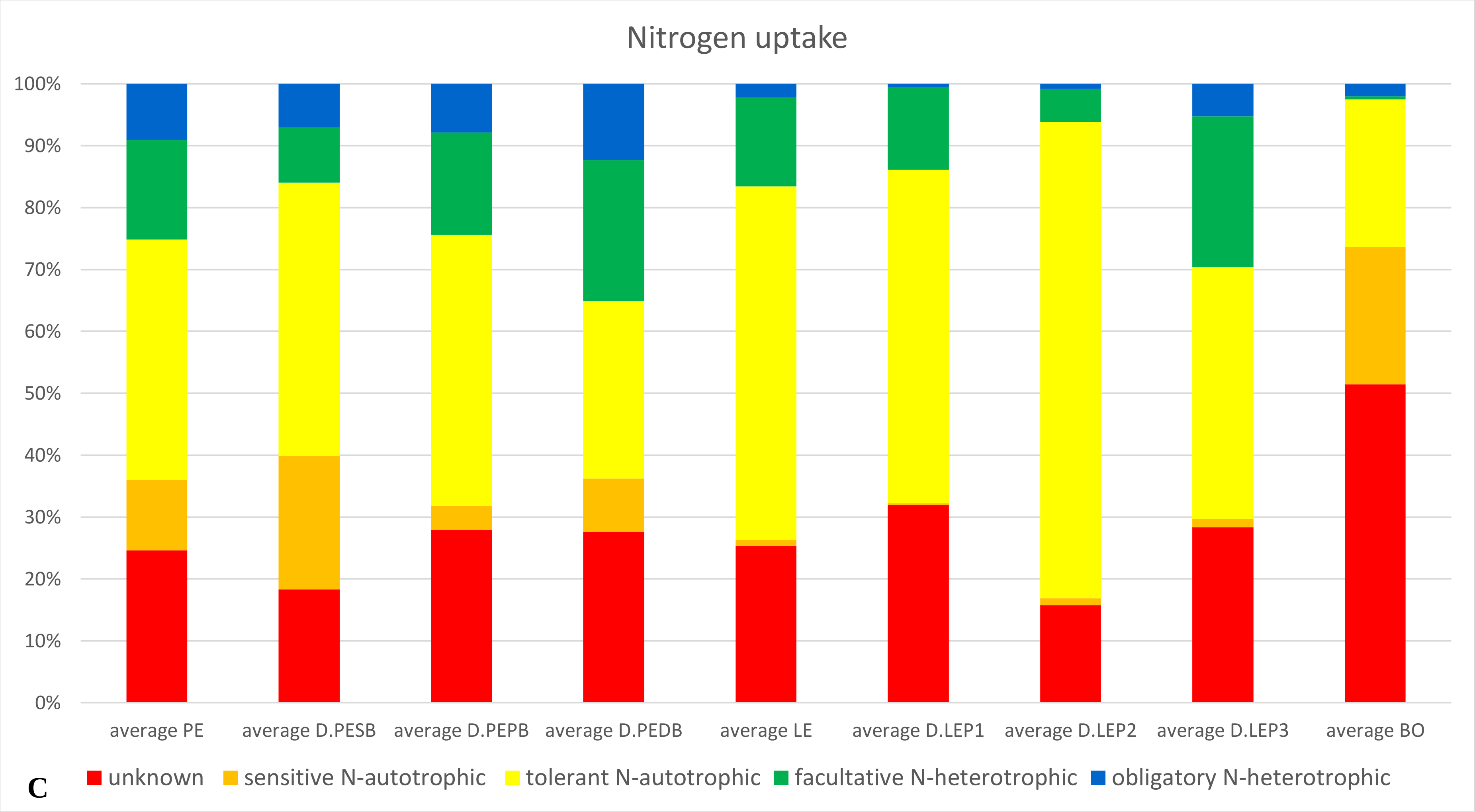
Nitrogen uptake
100%
90%
80%
70%
60%
50%
40%
30%
20%
10%
0%
average PE
average D.PESB
average D.PEPB
average D.PEDB
average LE
average D.LEP1
average D.LEP2
average D.LEP3
average BO
C
unknown
sensitive N-autotrophic
tolerant N-autotrophic
facultative N-heterotrophic
obligatory N-heterotrophic

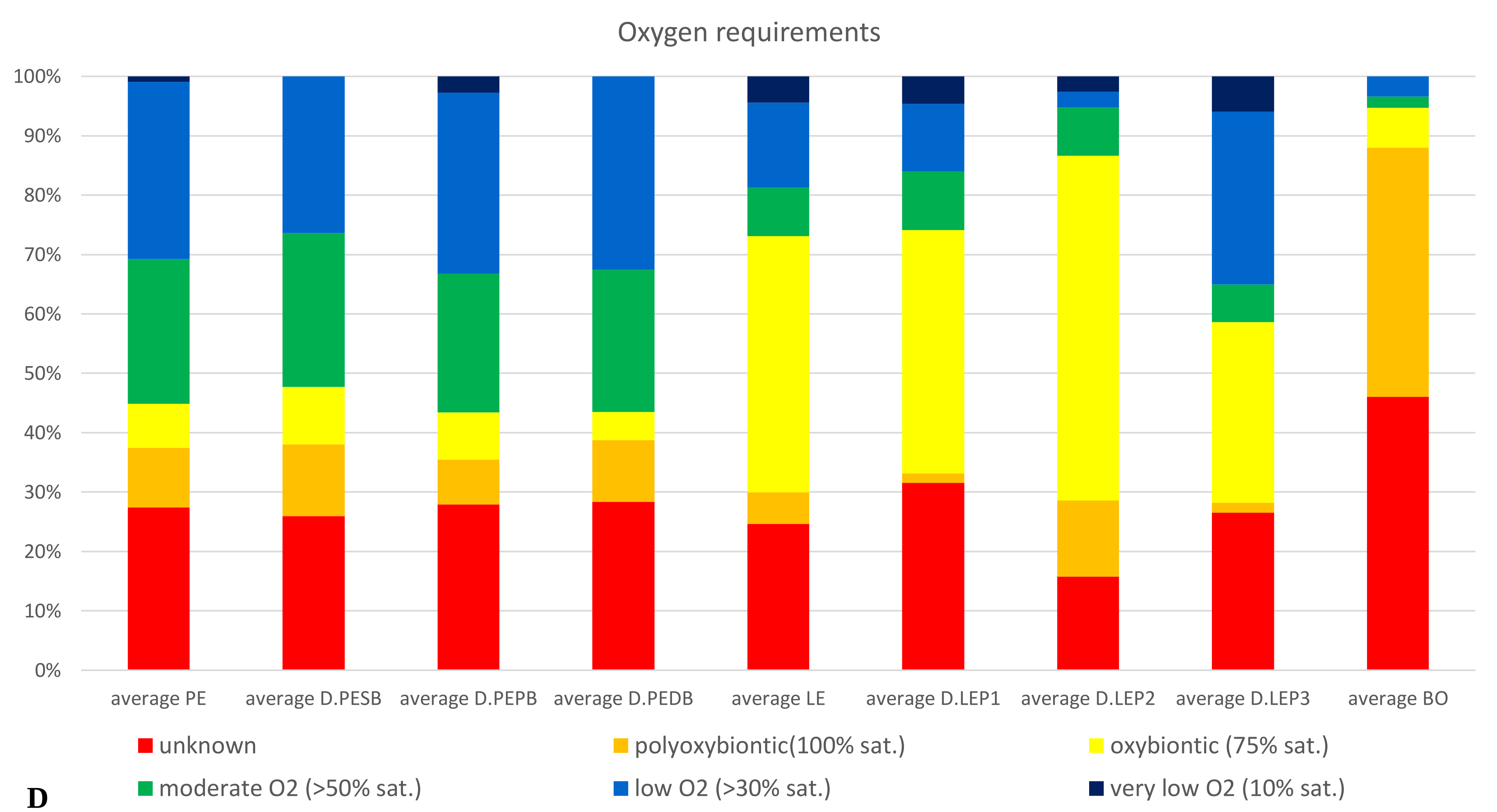
Oxygen requirements
100%
90%
80%
70%
60%
50%
40%
30%
20%
10%
0%
average PE
average D.PESB
average D.PEPB
average D.PEDB
average LE
average D.LEP1
average D.LEP2
average D.LEP3
average BO
unknown
polyoxybiontic(100% sat.)
oxybiontic (75% sat.)
moderate O2 (>50% sat.)
low O2 (>30% sat.)
very low O2 (10% sat.)

D

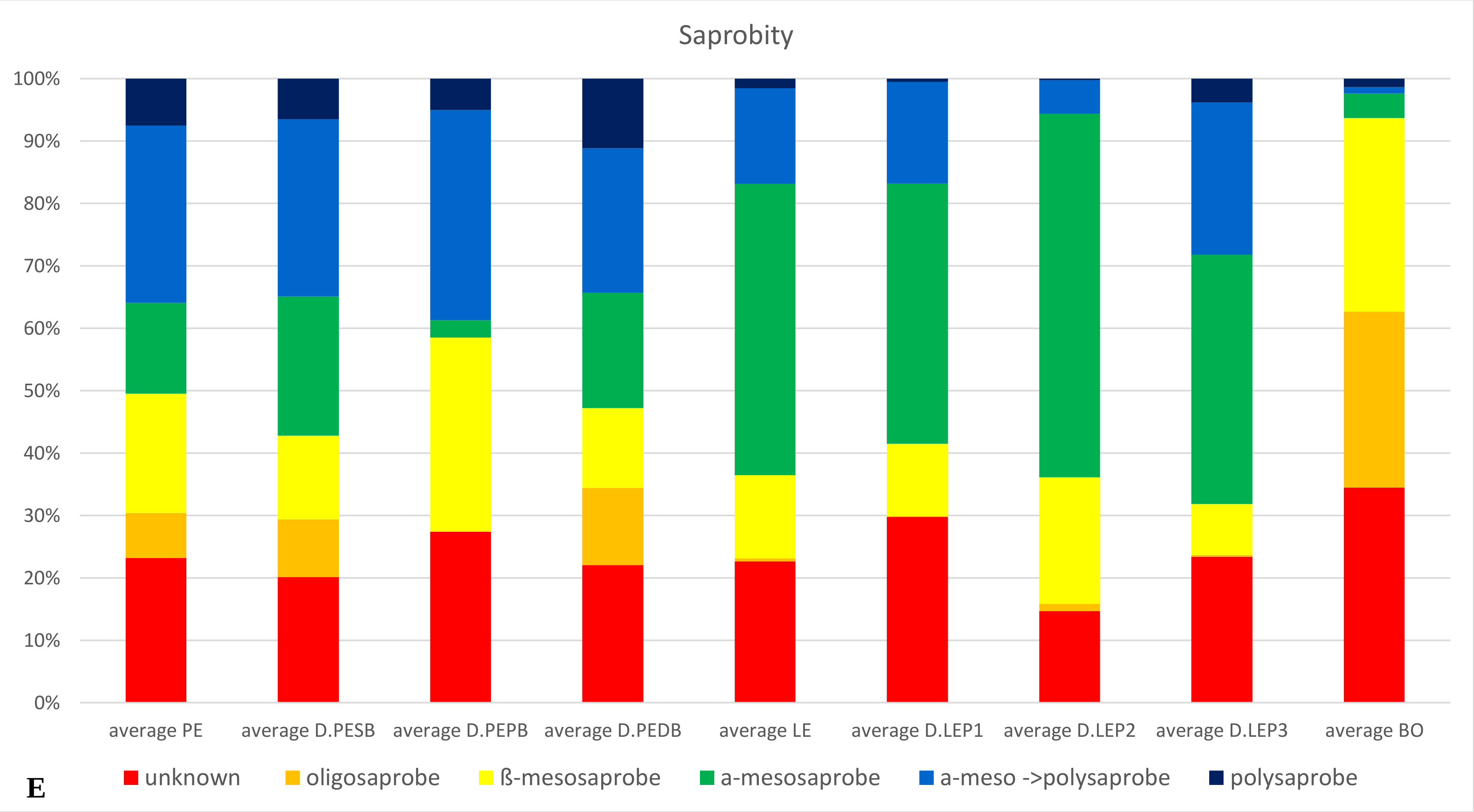

Saprobity
100%
90%
80%
70%
60%
50%
40%
30%
20%
10%
0%
average PE
average D.PESB
average D.PEPB
average D.PEDB
average LE
average D.LEP1
average D.LEP2
average D.LEP3
average BO
unknown
oligosaprobe
ß-mesosaprobe
a-mesosaprobe
a-meso ->polysaprobe
polysaprobe

E

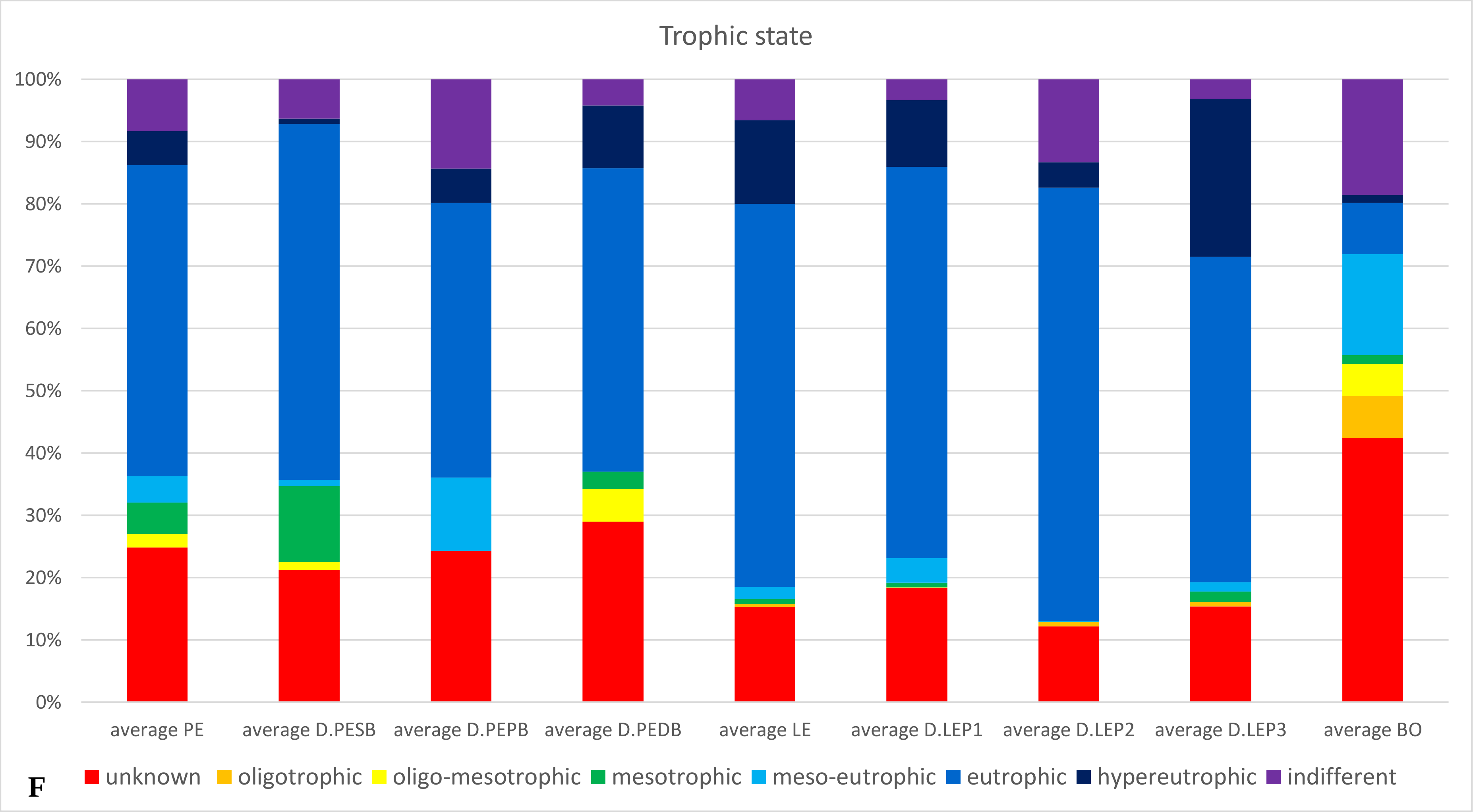
Trophic state
100%
90%
80%
70%
60%
50%
40%
30%
20%
10%
0%
average PE
average D.PESB
average D.PEPB
average D.PEDB
average LE
average D.LEP1
average D.LEP2
average D.LEP3
average BO
unknown
oligotrophic
oligo-mesotrophic
mesotrophic
meso-eutrophic
eutrophic
hypereutrophic
indifferent
F

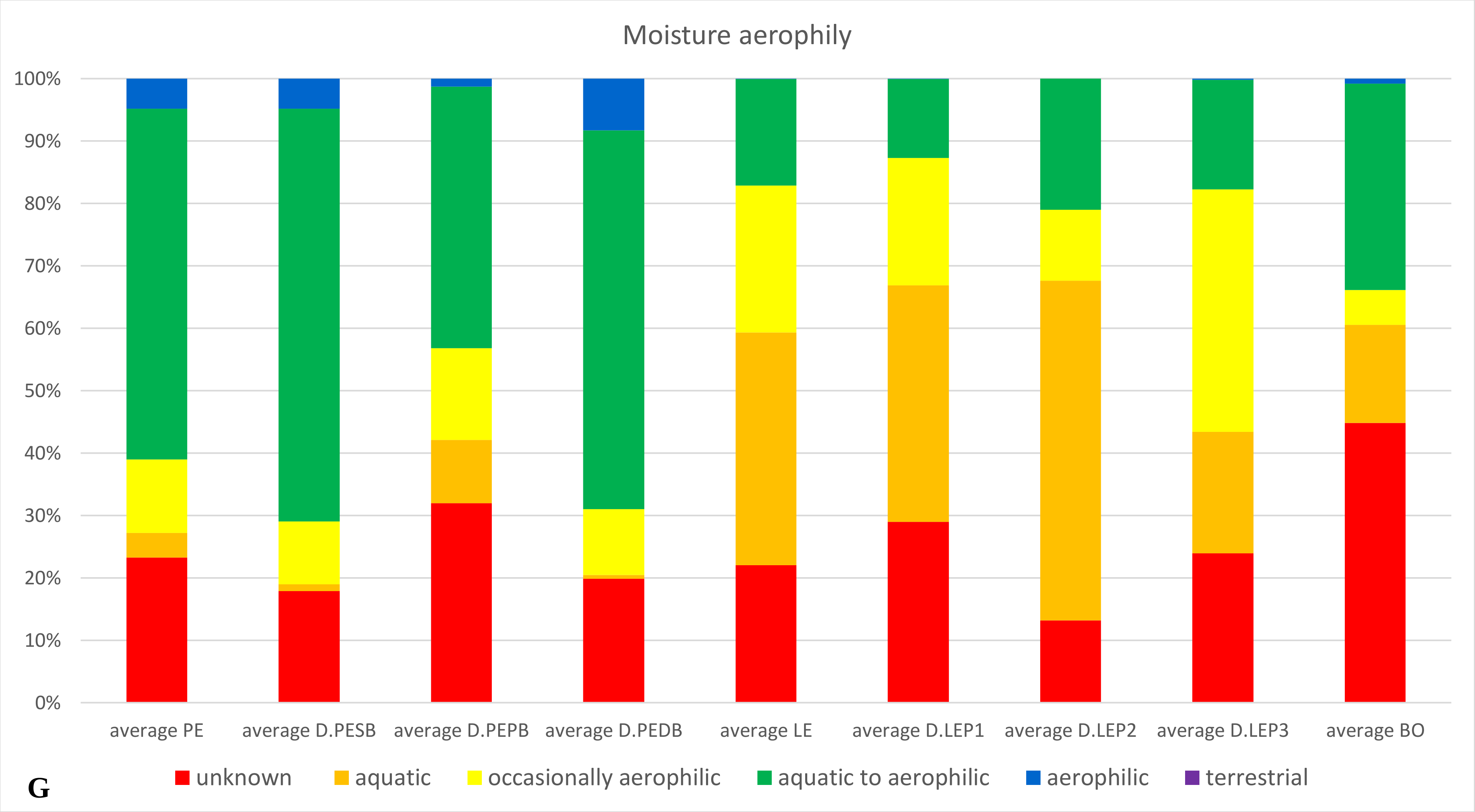
Moisture aerophily
100%
90%
80%
70%
60%
50%
40%
30%
20%
10%
0%
average PE
average D.PESB
average D.PEPB
average D.PEDB
average LE
average D.LEP1
average D.LEP2
average D.LEP3
average BO
unknown
aquatic
occasionally aerophilic
aquatic to aerophilic
aerophilic
terrestrial
G
