# Supplementary material for: Critical multi-stranded approach for determining the ecological values of diatoms in unique aquatic ecosystems of anthropogenic origin
Source: PeerJ. 2019 Dec 5;7:e8117. doi: 10.7717/peerj.8117 (PMC6899344; doi:10.7717/peerj.8117)
Supplement: Supplemental Information 3 [file peerj-07-8117-s003.docx]

| **Ecological parameters** |  | **Class (*Van Dam et al. 1994*)** | | | | | | | |
| --- | --- | --- | --- | --- | --- | --- | --- | --- | --- |
|  |  | 0 | 1 | 2 | 3 | 4 | 5 | 6 | 7 |
|  | pH requirements | unknown | acidobiontic | acidophilic | neutrophilic | alkaliphilic | alkalibiontic | indifferent |  |
|  | Salinity | unknown | halophobe | oligohalobous | halophilic | mesohalobous | brackish-marine | marine-brackish | marine |
|  | Nitrogen utake | unknown | sensitive  N-autotrophic | tolerant  N-autotrophic | facultative  N-autotrophic | obligatory  N-autotrophic |  |  |  |
|  | O_2_ requirements | unknown | polyoxybiontic | oxybiontic | moderate | low O_2_ | very low O_2_ |  |  |
|  | Saprobity | unknown | oligosaprobe | β-mesosaprobe | a-mesosaprobe | a-meso -> polysaprobe | polysaprobe |  |  |
|  | Trophy state | unknown | oligotrophic | oligo-mesotrophic | mesotrophic | meso-eutrophic | eutrophic | hypereutrophic | indifferent |
|  | Moisture aerophily | unknown | aquatic | occasionally aerophilic | aquatic to aerophilic | aerophilic | terrestial |  |  |
