# Supplementary material for: Critical multi-stranded approach for determining the ecological values of diatoms in unique aquatic ecosystems of anthropogenic origin
Source: PeerJ. 2019 Dec 5;7:e8117. doi: 10.7717/peerj.8117 (PMC6899344; doi:10.7717/peerj.8117)
Supplement: Supplemental Information 4 — Percentage of individual characteristic species constituting at least 10% of the ’unknown’ class at each sampling point. [file peerj-07-8117-s004.docx]

|  | pH | Salinity | Nitrogen uptake | Oxygen requirements | Saprobity | Trophic state | Moisture aerophily |
| --- | --- | --- | --- | --- | --- | --- | --- |
| D.PESB. | total 33% | total 44% | total 34% | total 48% | total 30% | total 22% | total 43% |
|  | CHPL (19%) NCCA (11%) | CHPL (33%) CHKF (10%) | CHPL (11%) NCCA (10%) | PTDE (25%) | CHPL (11%) | CHPL (10%) | PLFQ (20%) |
| D.PEDB. | total 92% | total 25% | total 82% | total 82% | total 80% | total 74% | total 85% |
|  | CHPL (60%) NCCA (32%) | CHPL (25%) | NLBT (37%) CHPL (25%) CHKF (16%) | NLBT (37%) CHPL (16%) CHKF (16%) | NLBT (43%) CHPL (32%) | NLBT (365) CHPL (23%) CHKF (15%) | NLBT (49%) CHPL (285) |
| D.PEPB. | total 81% | total 73% | total 78% | total 78% | total 79% | total 60% | total 84% |
|  | CRBU (53%) NCCA (27%) | CRBU (73%) | CRBU (46%) NCCA (25%) | CRBU (46%) NCCA (25%) | CRBU (46%) NCCA (26%) | CRBU (54%) | CRBU (41%) NCCA (23%) PLFQ (14%) |
| D.LEP1. | total 76% | total 79% | total 67% | total 68% | total 71% | total 54% | total 78% |
|  | CINV (36%) SBNT (23%) NMOK (16%) | SBNT (56%) NMOK (21%) | CINV (29%) SBNT (19%) NMOK (14%) | CINV (29%) SBNT (19%) NMOK (14%) | CINV (31%) SBNT (20%) NMOK (14%) | SBNT (36%) NMOK (17%) | CINV (32%) SBNT (22%) NMOK (14%) |
| D.LEP2. | total 39% | total 44% | total 35% | total 35% | total 28% | total 23% | total 44% |
|  | SBNT (27%) CINV (11%) | SBNT (44%) | SBNT (18%) | SBNT (18%) | SBNT (20%) | SBNT (23%) | SBNT (22%) SPAV (11%) CINV (11%) |
| D.LEP3. | total 57% | total 41% | total 50% | total 53% | total 60% | total 29% | total 57% |
|  | SBNT (25%) CINV (24%) | SBNT (41%) | SBNT (17%) CINV (15%) SPAV (13%) | SBNT (17%) CINV (16%) SPAV (14%) | SBNT (20%) CINV (18%) SPAV (15%) | SBNT (29%) | SBNT (20%) CINV (17%) SPAV (15%) |
| D.BOZB. | total 33% | total 36% | total 47% | total 44% | total 38% | total 47% | total 38% |
|  | PPCS (22%) DMOF (10%) | PPCS (25%) DMOF (11%) | MSMI (13%) PPCS (10%) | MSMI (14%) PPCS (11%) NDME (10%) | PPCS (18%) NDME (11%) | MSMI (15%) PPCS (12%) NDME (11%) | PPCS (13%) |
